# Lock, Stock and Barrel: Role of Renin-Angiotensin-Aldosterone System in Coronavirus Disease 2019

**DOI:** 10.3390/cells10071752

**Published:** 2021-07-11

**Authors:** Christian Zanza, Michele Fidel Tassi, Tatsiana Romenskaya, Fabio Piccolella, Ludovico Abenavoli, Francesco Franceschi, Andrea Piccioni, Veronica Ojetti, Angela Saviano, Barbara Canonico, Mariele Montanari, Loris Zamai, Marco Artico, Chiara Robba, Fabrizio Racca, Yaroslava Longhitano

**Affiliations:** 1 Department of Emergency Medicine, Foundation of Policlinico Agostino Gemelli-IRCCS, Catholic University of Sacred Heart, 00168 Rome, Italy; francesco.franceschi@unicatt.it (F.F.); andrea.piccioni@policlinicogemelli.it (A.P.); veronica.ojetti@unicatt.it (V.O.); saviange@libero.it (A.S.); 2 Department of Anesthesia and Critical Care, AON SS Antonio e Biagio e Cesare Arrigo, 15121 Alessandria, Italy; tatsiana_romenskaya@yahoo.it (T.R.); fpiccolella@ospedale.al.it (F.P.); fracca@ospedale.al.it (F.R.); lon.yaro@gmail.com (Y.L.); 3 Foundation Ospedale Alba-Bra and Department of Anesthesia, Critical Care and Emergency Medicine, Pietro and Michele Ferrero Hospital, 12051 Verduno, Italy; 4 Department of Emergency Medicine, AON SS Antonio e Biagio e Cesare Arrigo, 15121 Alessandria, Italy; michelefidel.tassi@gmail.com; 5 Department of Health Sciences, University Magna Graecia, 88100 Catanzaro, Italy; l.abenavoli@unicz.it; 6 Department of Biomolecular Sciences, University of Urbino Carlo Bo, 61029 Urbino, Italy; barbara.canonico@uniurb.it (B.C.); mariele.montanari@uniurb.it (M.M.); loris.zamai@uniurb.it (L.Z.); 7 National Institute for Nuclear Physics (INFN)-Gran Sasso National Laboratory (LNGS), 67100 Assergi L’Aquila, Italy; 8 Department of Sensory Organs, Sapienza University of Rome, 00185 Rome, Italy; marco.artico@uniroma1.it; 9 Department of Surgical Sciences and Integrated Diagnostics (DISC), University of Genoa, 16132 Genoa, Italy; kiarobba@gmail.com

**Keywords:** COVID-19, SARS-CoV2, ACE2, renin-angiotensin-aldosterone system, ACE inhibitors, sartans, zinc-chelating agents

## Abstract

Since the end of 2019, the medical-scientific community has been facing a terrible pandemic caused by a new airborne viral agent known as SARS-CoV2. Already in the early stages of the pandemic, following the discovery that the virus uses the ACE2 cell receptor as a molecular target to infect the cells of our body, it was hypothesized that the renin-angiotensin-aldosterone system was involved in the pathogenesis of the disease. Since then, numerous studies have been published on the subject, but the exact role of the renin-angiotensin-aldosterone system in the pathogenesis of COVID-19 is still a matter of debate. RAAS represents an important protagonist in the pathogenesis of COVID-19, providing the virus with the receptor of entry into host cells and determining its organotropism. Furthermore, following infection, the virus is able to cause an increase in plasma ACE2 activity, compromising the normal function of the RAAS. This dysfunction could contribute to the establishment of the thrombo-inflammatory state characteristic of severe forms of COVID-19. Drugs targeting RAAS represent promising therapeutic options for COVID-19 sufferers.

## 1. Introduction

From the end of the year 2019, the world is struggling for a deadly pandemic used by a new emerging viral agent, known as SARS-Coronavirus 2 (SARS-CoV2), which have caused almost 2.5 million deaths so far [1].

Since the very beginning of the global emergency, the scientific community put all its effort to better understand the nature of the new pathogen, providing more and more information about the structure, the vital cycle and the pathophysiology of the virus.

It was soon evident that, likewise its predecessor SARS-CoV1, responsible for the 2002 outbreak, SARS-CoV2 is able to infect our cells thanks to the interaction with angiotensin converting enzyme II (ACE2) [2,3,4], a recently discovered transmembrane glycoprotein with enzymatic activity, belonging to the renin-angiotensin-aldosterone system (RAAS). Given these premises, many research groups set themselves the goal of understanding whether and to what extent the renin-angiotensin-aldosterone system was involved in the pathogenesis of Coronavirus disease 2019 (COVID-19).

In this article, more than a year after the beginning of the pandemic, we review the current literature on the subject, trying to clarify what we think to know, what we are still trying to understand and where this can lead us.

## 2. Renin-Angiotensin-Aldosterone System

RAAS is an endocrine and paracrine system widely expressed across different body tissues and essential for the maintenance of several homeostatic functions, such as blood pressure, fluid and electrolytes balance and local tissues perfusion.

First element in the system, renin, is actually the active enzymatic form of the zymogen prorenin, produced by the kidney granular cells in response to various triggers, such as low blood pressure, low sodium concentration and sympathetic stimulation [5,6,7]; prorenin is then either proteolytically activated directly into the kidney by neuroendocrine convertase 1 and cathepsin B, or released in the blood and then non-proteolytically activated by tissues expressing prorenin-receptor [6]. Once in its active form, renin is able to bind and hydrolyse the circulating, liver secreted, alfa2-globulin angiotensinogen, generating angiotensin I (AT1) which is further cleaved by another membrane protease, named angiotensin converting enzyme (ACE), exposed by the endothelial cells in lung and other organs’ vascular beds [8,9]. Final product of this proteolytic cascade, angiotensin II (AT2) acts by binding to angiotensin 2 receptors (AT2Rs), a group of G proteins coupled receptors with different subtypes, that allow AT2 to elicit pleiotropic effects in human body [6,10,11]: it produces arteriolar and venous vasoconstriction, raising blood pressure and heart rate; it stimulates the adrenal cortex to release aldosterone, which in turns, acting on the distal tubules in the kidney, produces sodium retention and potassium loss; in the central nervous system, it acts on the hypothalamus promoting thirst sensation, antidiuretic hormone secretion and—as a result-fluid retention and hypertension. In addition, AT2 might promote lipogenesis, thus increasing adipose tissue mass [12].

Moreover, beside the rapid effects mediated by G proteins, the AT2 type 1 receptor (AT2R1) is able to activate intracellular pathways that lead to the production of transcription factors and mediators involved in the genesis of several pathophysiological processes.

First of all, AT2 via AT2 type 1 receptors determines the activation of intracellular signaling systems, including the nuclear factor-κB (NF-κB), mitogen-activated protein kinase (MAPK) cascade and Rho proteins, that lead to the upregulation of a multitude of pro-inflammatory genes [10,13,14,15,16,17]. In fact, AT2 promotes numerous key steps of the inflammatory process such as the increase in vascular permeability, through the release of VEGF, and the recruitment of inflammatory cells, through the expression of cytokines and chemokines such as IL-6 and MCP-1, as well as adhesion molecules such as VCAM-1, ICAM-1 and integrins [16]. Furthermore, by determining the activation of NADPH-oxidase, AT2 stimulates the production of ROS [18,19].

Secondly, the activation of local RAAS systems is able to produce prothrombotic effects: in fact, AT2 increases the expression of platelet adhesion molecules, tissue factor and tissue plasminogen activator inhibitor (PAI-1) [20].

In addition, the RAAS system takes part in the processes of tissue fibrosis and hypertrophy. Evidence indicates that AT2 via AT2R1 regulates extracellular matrix (ECM) accumulation mediated by the endogenous production of profibrotic growth factors such as fibroblast growth factors (FGF), platelet-derived growth factor (PDGF), vascular endothelial growth factor (VEGF) and transforming growth factor-beta (TGF-beta) [21,22,23]. In addition, AT2 upregulates connective tissue growth factor (CTGF), the downstream mediator of TGF-beta [22].

RAAS-mediated inflammation and fibrosis have been implicated in a multitude of chronic pathologies, such as hypertension, metabolic syndrome, ischemic heart disease, heart failure, chronic kidney disease, steatohepatitis and neurodegenerative diseases [6,24]. This knowledge led almost half a century ago to the development of ACE-inhibitors (ACEIs) and, later on, angiotensin receptor blockers (ARBs, also called sartans). In addition to their antihypertensive properties, these drugs have demonstrated to slow down disease progression and prolong survival in many of the above conditions, thanks to their ability to reduce AT2-related inflammation and fibrosis [25].

In an evolutionary perspective, given RAAS central role in human physiology and the consequences his dysfunction can lead to, it is natural for its activity to be finely regulated both at the systemic and local level. As already described, renin release is strictly regulated in response to perturbations in blood pressure, acid-base status and hydroelectrolytic homeostasis; plasma level of angiotensinogen can be increased by corticosteroid, estrogen, thyroid hormone and AT2 levels [6]; aldosterone secretion is stimulated not only by AT2 but also by ACTH, increase in plasma potassium level and acidosis [25].

In addition, AT2R1 is not the only AT2 active target, as this hormone can interact with at least one other receptor types, namely AT2R2 [26,27,28]. This receptor is usually thought to mediate anti-inflammatory effect by inducing NO production and by activating several signaling pathways, such as intracellular phosphatases and PI3K, that inhibit MAPK and NF-Kb, thus mitigating many of the effects of AT2R1 activation. However, AT2R2 role is still controversial, as other studies showed that it can also mediate pro-inflammatory effects [29].

**Figure 1:**
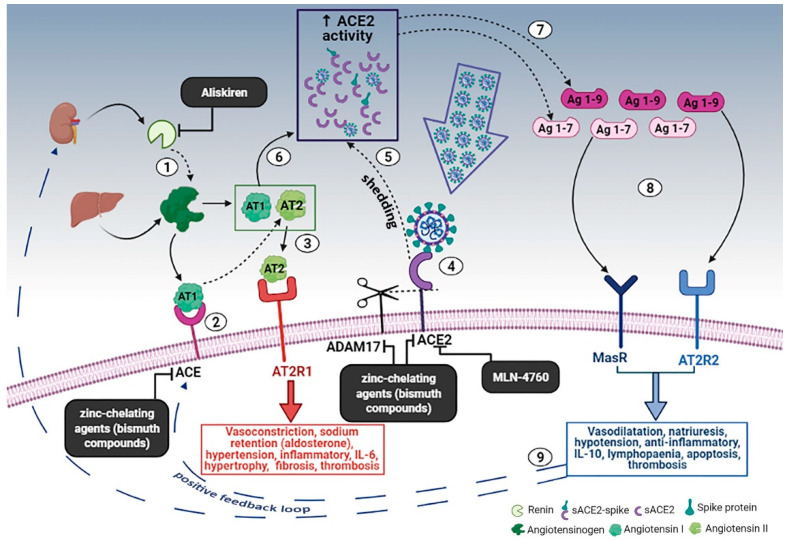
Schematic diagram of the effects of RAAS system during SARS-CoV-2 infection and the proposed therapeutic treatment. (1) Renin secreted, by the kidney, cleaves angiotensinogen, produced by liver, to form AT1, (2) AT1 is converted to AT2 by pulmonary ACE. (3) AT2 binds to AT2R1 (Angiotensin II receptors 1). The excess of AT2 through AT2R1 hyperactivation causes vasoconstriction, sodium retention (by aldosterone release), hypertension, inflammatory, IL-6, hypertrophy, fibrosis, thrombosis. (4) SARS-CoV-2 binds to ACE2 to gain entry into the host cell, however, cellular protective response leads to ACE2 shedding. (5) ADAM17-regulated ectodomain shedding of ACE2 results in increased amount of soluble and active ACE2 (sACE2). (6) AT1 and AT2 can also bind to sACE2. (7) They are then metabolized by ACE2 into Ag 1–9 and Ag 1–7, respectively. (8) The excess of Ag 1–9 and Ag 1–7 signaling via the AT2R2 and MasR can induce vasodilatation, natriuresis, hypotension, anti-inflammatory, IL-10, lymphopenia, apoptosis, thrombosis. (9) These events, in turn, produce a compensatory upregulation of both renin secretion and ACE activity, which establish the onset of a positive feedback loop. In the black boxes, drugs that can potentially to stop the positive feedback loop, by inhibiting enzymes of the RAAS, are indicated. Dashed arrows indicate enzymatic activity, full arrows indicate non enzymatic passage and dashed blue arrows represent the positive feedback loop. Created in Biorender.com.

Furthermore, in the last decades it was discovered the existence of a RAAS counter-regulatory system headed by another membrane protein, named angiotensin converting enzyme 2 (ACE2) [6,27,28,30]. Similarly, to ACE, ACE2 is a transmembrane glycoprotein with an extracellular enzymatic domain; it is almost ubiquitous expressed in human organs but is localized in specific cell types in many tissues and its level and activity vary in response to various local and systemic stimulus (see later). ACE2 cleaves circulating angiotensin I and II to generate several active peptides, among them Ag 1–7 and Ag 1–9, with different properties: by interacting with AT2R2 and MasR receptors, these peptides have shown to produce vasodilatation, reduce blood pressure, promote natriuresis and inhibit thrombotic, flogistic and fibrotic processes, counteracting as a result the effects produced by the activation of AT2R1 by AT2 [27,28,30] (Figure 1).

Hence, it is now believed that effects of RAAS activation depend on the tissue ACE/ACE2 balance, which determines the availability of different angiotensin peptides and hence the balance between pro-inflammatory and pro-fibrotic, and anti-inflammatory and anti-fibrotic pathways.

## 3. Virology of SARS-CoV2

**Figure 2:**
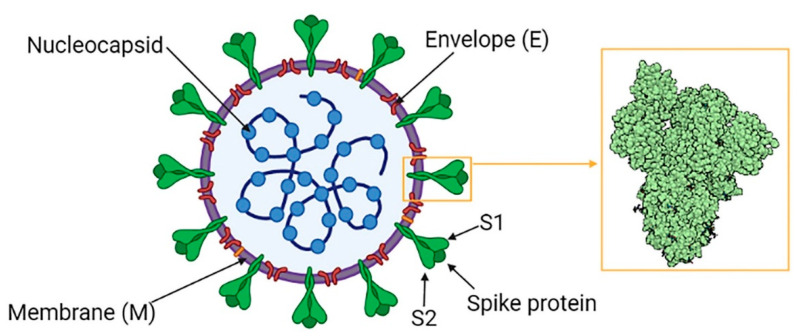
SARS-CoV 2 Structure. In the yellow box the structure-based design of prefusion-stabilized SARS-CoV-2 spikes. Created in Biorender.com.

SARS-CoV 2 is a member of Coronaviridae, a family of enveloped positive-sense single-stranded RNA viruses, named after their characteristic appearance under the electron microscope which resembles that of a crown. Together with its relatives SARS-CoV1 and MERS-CoV, SARS-CoV 2 belongs to the β-CoV genre in the Coronavirinae subfamily [31]. It is composed of a nucleocapsid (protein N)—which contains the viral genome—and an envelope formed by viral structural protein S (spike), E (envelope) and M (membrane) inserted in a double layer phospholipid membrane (Figure 2) [32].

Coronavirus spike proteins are responsible for viral binding to host cells, as they act as class I membrane fusion proteins; they contain a receptor binding domain (RBD), which can recognize various cell surface targets, including proteins, carbohydrates and heparan sulfate. Several receptors were evaluated as potential target of SARS-CoV2 infection, such as DPP4 (dipeptidyl peptidase 4, already know to be MERS-CoV receptor), KIM1 (kidney injury molecules 1), NRP1 (neuropilin-1) and cluster of differentiation 147 (an extracellular matrix-metalloprotease expressed on erythrocytes, leucocytes, platelets and endothelial cells) [33,34,35,36,37,38]. Nevertheless, it is likely that, similarly to its predecessor SARS-CoV1, SARS-CoV2 uses ACE2 as his main receptor to infect human cells [3,4,39]. It was also proved that SARS-CoV2 affinity for ACE2 receptors is 10–20 times higher than the one shown by SARS-CoV1 [4], a fact that can potentially explain its more infective potential.

The mechanisms used by the virus to enter the cell and replicate itself was also studied [2,4,39,40,41]: the interaction between RBD on the S1 subunit of SARS-CoV2 spike proteins and ACE2 receptors on the target cell membrane, provokes a conformational shift in protein S which promotes viral endocytosis and exposes a short amino acid sequence at the S1/S2 junction; this domain seems capable of interacting with several cellular protease families, including furins, cathepsins, trypsins and type II transmembrane serine proteases (TTSP) [42], allowing the fusion between the viral envelope and the plasma membrane; the S2 subunit then undergoes proteolytic priming, releasing the virus into the cytosol. Among the various protease, TMPRSS2 seems to play a central role, as it is co-expressed along with ACE2 receptors in many tissues targeted by the virus, and inhibitors of TMPRSS2 are proved to prevent viral entry into the cell in vitro [2,43,44].

SARS-Cov2 genomic RNA contains a 5’ CAP and a 3’ poly(A) tail which allows it to be directly translated by cell ribosome in the RER (rough endoplasmic reticulum) to produce two replicase polyproteins (pp1a and pp1ab) that are then cleaved by the action of viral proteases nsp3-PLpro and nsp5-Mpro into nonstructural proteins (nsps) [31,41]. Nonstructural proteins, in turn, promote rough endoplasmic reticulum (RER) rearrangement to generate double membrane vesicles and are further assembled to form the replicase-transcriptase complex, essential to produce anti-sense copies of viral genome which are used as templates to synthesize positive-sense genome and structural proteins encoding mRNA. Newly synthesized structural proteins are inserted in the RER membrane and pass through the secretory ER-Golgi apparatus pathway, while positive-sense copies of the viral genome form a complex with N proteins. So-formed virions are eventually transported to the cell surface inside vesicles and exocytosed [41].

## 4. ACE2 and Organ Tropism of SARS-CoV2

SARS-CoV2 is transmitted by air through respiratory droplets. In fact, the main gate for the virus to enter and leave the human body is represented by the airways [45,46,47].

The natural history of disease shows a triphasic course: COVID-19 usually begins as a influenza-like syndrome with fever, dry cough, throat pain, fatigue, arthromyalgia and GI symptoms. Typical is also the involvement of olfactory and taste sensations, with anosmia and ageusia. In about 80% of cases, the disease has a benign course and these symptoms are the sole manifestations of the infection; on the contrary, around 20% of patients develop clinical evident pneumonia, characterized by dyspnea, chest pain, reduced blood oxygen and bilateral lung infiltrates on chest imaging. In some of these patients, the disease further progresses with the development of a systemic hyperinflammatory state that leads to ARDS, thrombotic manifestations, multi-organ damage and eventually death [40].

At the beginning of infection, SARS-CoV2 may pass through the nasal and pharyngeal mucosa or directly affect the lower respiratory tract, infecting bronchial and alveolar cells. ACE2 receptor was found in nasal and bronchial epithelium, and is also highly expressed by type II pneumocytes [47,48,49]; the latter also co-express transmembrane protease serine 2 (TMPRSS2), which, as already mentioned, is believed to be crucial for viral entry and replication [2,43].

Type 2 pneumocytes are essential cells for the maintenance of lung function: in fact they produce the so-called surfactant [25,47], a mixture of phospholipids which, by reducing the alveolar surface tension, prevents their collapse and promotes respiratory exchanges; they also possess immune-modulatory functions and can proliferate and differentiate into type 1 pneumocytes, guaranteeing their natural turnover. At the basement membrane level, type 2 pneumocytes border directly with the endothelial cells of the pulmonary microcirculation, which in turn express ACE-2 receptors as well as TMPRSS2 [50,51]. For all this reasons, it is believed that these cells represent a fundamental actor in the pathogenesis of lung damage during COVID-19 [45,47,52,53,54]: the cytotoxic effects produced by the infection and replication of the virus in type II pneumocytes would determine their apoptosis, as well as the production of cytokines and chemokines with the recall of a massive cellular infiltrate of monocyte-macrophages and neutrophils and further release of cytotoxic and pro-inflammatory factors, in an actual cytokine storm. To this regard, endothelial dysfunction represents another key player in the development of COVID-19 [55,56,57]: imaging and isthopathological findings demonstrated vascular damage and flogosis (vasculitis) in affected lungs, along with microvascular thrombosis; this is due to the fact that SARS-CoV2 is capable of determine endothelial damage either directly, by infecting the endothelium, with consequent cellular dysfunction and apoptosis or indirectly via cytokine release. This would lead to impairment of the normal function of the pulmonary microcirculation, promoting thrombosis, further pro-inflammatory cytokine production and loss of alveolar-capillary barrier integrity. Subsequently, the activation of cellular repair mechanisms would lead to hyperplasia of type 2 pneumocytes and proliferation of fibroblasts with remodeling of the pulmonary interstitium. This would result in the picture of diffuse alveolar damage, fibrinous exudate, hyaline membranes formation and, in the most advanced forms, interstitial fibrosis, observed in autopsy studies on patients who died from COVID-19 [58,59,60,61,62]. At the same time, the cytokine storm would have repercussions on the whole organism, predisposing to thrombotic phenomena and favoring the onset of multi-organ damage.

Additionally, in the initial phase, the virus can enter the bloodstream via the lungs endothelial cells and then proceed to affect other organs expressing ACE2. Accordingly, in autopsy studies on COVID-19 patients, the presence of the virus has been demonstrated in many organs other than the lungs, such as the GI tract, kidneys, heart, liver and brain [63]. However, viral particles are rarely detected in blood samples from infected patients [64,65,66], suggesting that viremia is not the primary mechanism for viral spreading through the body. An alternative diffusion route may be represented by replication into the reticular-endothelial system, as alveolar macrophage seems able to uptake, amplificate and then release the virus [67].

Several studies have shown that ACE2 is highly expressed in the epithelial cells of the esophagus and in the brush border of enterocytes, where it is co-expressed with TMPRSS2 [47,68]. Not surprisingly, many patients with COVID-19 show signs of involvement of the digestive tract already in the early stages of the disease. Gastrointestinal symptoms have been reported in up to 17% of patients [69], and include inappetence, diarrhea, nausea, vomiting and abdominal pain. Copies of viral RNA are detectable in the feces in up to half of COVID-19 patients, even in cases with undetectable viral RNA in airway samples [70,71]. Therefore, it is believed that SARS-CoV2 is able to infect intestinal cells directly or following hematogenous dissemination, to the point that some authors have invited to consider the fecal-oral route as a possible secondary route of viral transmission.

In the kidneys, ACE2 is expressed mostly in the brush borders of the proximal tubular epithelium and in podocytes, at the glomerular level [72]. Accordingly, histopathological analyses show that COVID-19 patients can develop diffuse proximal tubule damage, with presence of virus-like particles in tubular epithelial cells and podocytes [73,74,75]. Alternatively, it was proposed that SARS-CoV2 may also be able to interact with a different receptor in the kidneys, namely kidney injury molecule 1 (KIM1), which is overexpressed during renal damage [34,35]. These findings could account for the clinical picture of acute kidney injury and proteinuria which affects up to 15% of COVID-19 patients [76].

Cardiac involvement is a prominent feature in COVID-19 and is associated with a worse prognosis [77,78]. Acute myocardial injury, in the form of increased levels of high-sensitive Troponin I, can be detected in up to 28% of patients [79,80]. Several mechanisms have been proposed, including oxygen delivery- demand imbalance due to hypoxia, atherosclerotic plaque rupture in the context of a hypercoagulatory state, cytokine storm-induced damage and myocarditis secondary to either inflammation, autoimmunity or direct infection by the virus [81,82]. Even though the last mechanism is still a matter of debate, it is known that ACE2 is expressed in heart tissues [83], especially by pericytes as well as in the endothelium, and presence of viral RNA has been demonstrated in cardiac cells [63]; furthermore, some authors reported presence of interstitial inflammatory infiltrates, with monocytes-macrophages containing viral-like particles [84].

Signs of mild to moderate liver damage, such as elevated liver function tests (AST, ALT, γ-GT and ALP), hypoalbuminemia and prolonged prothrombin time, are also frequently reported in COVID-19 patients and histopathological alteration in liver tissue of deceased patient were reported [85,86]. Direct viral infection of perivenular hepatocytes and endothelial cells, the main ACE2-expressing cells in the liver, cannot be ruled out [87]. In this regard, hypoxic conditions upregulate ACE2 protein expression on human hepatocytes [88], thus increasing liver susceptibility to virus infection that could lead to a liver dysfunction and possibly to a liver-mediated hypercoagulatory state [27,89].

Last but not least, there are clues of a potential involvement of the brain by the virus [90,91]. Neurological abnormalities have been described in more than one third of patients [92], including malaise, dizziness, headache, alterations in mental status (confusion, disorientation, agitation and somnolence) and loss of smell and taste; also, ischemic stroke and encephalitis have been described [93,94,95]. Brains of deceased COVID-19 patients showed evidence of oedema and partial neuronal degeneration [85], white matter lesions [94] and direct presence of the SARS-CoV-2 in the brain has been reported [63,96], although other studies were not able to confirm this finding. There are two primary pathways by which SARS-CoV-2 may reach the central nervous system (CNS):

1. The virus may travel by retrograde axonal transport through sensory and olfactory nerves [97]. This pathway could account for COVID-19 related anosmia and ageusia.
2. An alternative route is through the dissemination of SARS-CoV-2 into the systemic circulation following infection of the respiratory tract [98].

In the CNS, ACE2 is expressed by neurons and glial cells, particularly in the brainstem and cardiovascular regulatory areas, such as nucleus of tractus solitarius, paraventricular nucleus and the rostral ventrolateral medulla [99,100]. Previous studies showed that SARS-CoV1 and MERS-CoV directly infect the brainstem [101]. It was hypothesized that the respiratory breakdown in COVID-19 patients may be at least in part caused by SARS-CoV-2 infecting respiratory centers in the medulla oblongata and the pons [90,91]. It was also proposed that neuropilin-1 (NRP1), a transmembrane receptor expressed in the respiratory and olfactory epithelium as well as in the CNS endothelial cells, may serve as an alternative cell receptor and facilitate SARS-CoV2 entry into the brain [36,37,102].

## 5. RAAS Dysfunction in COVID-19 Pathogenesis

According to various authors, the role of RAAS in the pathogenesis of COVID-19 is not limited to providing the virus with an access path to cells. In fact, there are numerous observations that suggest that a dysregulation of the tissue ACE/ACE2 balance during the course of the infection can contribute to the onset of organ damage and hyperinflammatory state.

For example, numerous conditions known to increase the risk of infection and/or disease severity, such as age, smoking, exposure to environmental pollutants, hypertension, obesity, diabetes, lung, liver, kidney and heart disease, are characterized by an increased activity in the canonical ACE-AgII-AT2R1 axis [103,104,105,106,107,108,109,110,111,112,113,114,115]. At the same time, in several of these conditions, the circulating ACE2 activity also increases [88,116,117].

Furthermore, it was observed that, after binding to the cell, SARS-CoV2 can promote the shedding of transmembrane ACE2 receptors by stimulating ADAM17 activity [45,46], with concomitant increase of the soluble circulating form (sACE2) levels. Thus, several authors hypothesize that a down-modulation of ACE2 tissue activity-due to its shedding or internalization into the cells along with the virus—and the subsequent ACE/ACE2 imbalance could substantially contribute to the genesis of the hyperinflammatory state seen in COVID-19 [47,48]. However, it was demonstrated that sACE2 retains not only the capability of binding the virus, forming circulating sACE2-spike protein complexes, but also its enzymatic activity [45,52,53], and that in COVID-19 patients circulating ACE2 activity is actually increased, correlates positively with disease severity and remain elevated even after disease resolution [53,118,119,120,121].

For these reasons, other authors suggested that, at the systemic level, SARS-CoV2 induces ACE2 up-modulation and that such an up-modulation represents a co-factor in the development of severe disease [27,89]. This statement may sound contradictory, as ACE2 activity is usually thought to mediate beneficial effects in many pathological states through its vasodilatory, natriuretic and anti-inflammatory properties [27,28,30]. However, ACE2 protective effects were usually observed in models where canonical ACE pathway was upregulated or ACE2 itself was downregulated, therefore balancing an unbalanced situation. In different models, (s)ACE2, Ag (1–7) or Ag (1–9) upregulation has been associated to pathological conditions such as inflammation of the kidneys and gastrointestinal tract, cardiac dysfunction, human cirrhosis, lung injury/fibrosis and microvascular thrombosis [59,60,61,62,68,107,108,122,123,124,125]; also, Ag (1–7) antiproliferative and apoptotic effects, possibly in part through IL-10 upregulation [110], may mediate eosinopenia and lymphopenia (two features commonly seen in COVID-19 patients), potentially impairing immune system ability to counteract viral infection [126,127,128,129]. Of note, IL-10 is significantly upregulated in the most severe forms of COVID-19 [130,131,132]. On the other hand, Ag (1–9), has been shown to enhance venous thrombosis mediated by fibrinolytic impairment [123] and that it’s receptor AT2R2 can mediate pro-inflammatory effects under certain circumstances [29]. Furthermore, it is known that hypoxia in combination or not with hypercapnia, can upregulate the activity of both arms of the renin–angiotensin system by inducing renin, ACE and ACE2 synthesis, which in turn can increase expression of AT1, AT2, Ag (1–7) and Ag (1–9) [123,132,133,134,135]. Accordingly, it was observed that, in lung aspirates of acid- and/or spike-treated mice, AT2 and ACE2 are synergistically upregulated and cell surface downregulated (shed), respectively, suggesting their involvement in the increased lung microvascular permeability and pulmonary oedema [136,137]. Finally, recent experimental data show that sACE2 may also contribute to viral spreading into the body. In fact, in vitro, sACE2-spike protein complex was able to enter cells directly through receptor-mediated endocytosis via the AT1 surface receptor, or indirectly through the interaction with vasopressin and the formation of an sACE2- sACE2-spike protein-vasopressin complex, which can bind to vasopressin receptor AVPR1B allowing cell infection [138]; of note ACE2 is one of the key regulators controlling the release of vasopressin into the plasma [139]. Accordingly with these findings, administration of recombinant ACE2 (rACE2) was seen to facilitate infection in vitro as well as in vivo [118,138].

In conclusion, it is likely that enhancement of both the canonical and non-canonical RAAS pathways by SARS-CoV2 infection, especially in subjects in which both arms of the system are already upregulated due to comorbidities, contributes to the development of acute lung injury, acute respiratory distress syndrome (ARDS) and other organ dysfunctions characteristics of severe COVID-19 forms.

## 6. RAAS Targeting Drugs and COVID-19

As it was discovered that ACE2 is the receptor for SARS-CoV2 entry into the cells, many authors raised a concern for the potential negative interactions between RAAS-targeting drugs (ACEI and ARBs) and SARS-Cov2: in fact, previous studies found that these medications may increase ACE2 levels, a fact that could potentially increase individual susceptibility to infection. On the other hand, some authors suggested potential benefits from such a therapy, advocating that inhibition of ACE-AT2-AT2R1 downstream pathways could possibly mitigate the thrombo-inflammatory state seen in severe COVID-19 cases.

The scientific community answered the call, and a multitude of researchers investigated the role of these medications in Coronavirus disease, with various results.

First preliminary data on RAAS-targeting drugs and COVID-19 interactions came from Chinese papers. In a retrospective cohort study that included 78 COVID-19 patients with hypertension, Liu et al. didn’t observe associations between previous treatment with any cardiovascular medication class and severe COVID-19, but the risk of severe COVID-19 in a subgroup of 46 patients older than 65 was lower in those who had received previous treatment with an ARB [140]. In a small retrospective study on 126 hypertensive COVID-19 patients in the province of Wuhan, Guang and colleagues found significantly lower C Reactive Protein (CRP) and procalcitonin levels in patients on ACE-inhibitors or ARBs therapy, as well as a non-significant trend toward reduced incidence of severe disease (9.3% vs. 22.9%; *p* = 0.061) and death (4.7% vs. 13.3% *p* = 0.283) [141]. Similar results were obtained by Meng and colleagues [142].

As the pandemic spread all around the world and the number of cases exponentially grew, more and more data on the subject became available. A large retrospective analysis [143], published in May 2020 on the NEJM, involving a total of 12,594 patients tested for SARS-CoV2 in New York City, explored the relations between the use of several anti-hypertensive drugs (ACE-inhibitors, ARBs, calcium channel blockers, beta-blockers, thiazide diuretics) and the risk of infection or severe illness. Previous treatment with drugs acting on RAAS was not associated with a higher risk of testing positive for COVID-19, neither was any of the above-mentioned medications associated with a substantial increase in the risk of severe illness among patients who tested positive.

In the same month, NEJM published the results of another multicentre observational study by Mandeep R. Mehra et al. [144]: in a cohort of 8910 hospitalized COVID-19 patients, use of ACE inhibitors was associated with better survival (odds ratio for mortality 0.33; 95% CI 0.20–0.54). However, the paper was then retired by the publisher because the authors did not grant access to the raw data of the study.

The positive results on RAAS targeting drugs in COVID-19 were further reinforced by the meta-analysis of Lu Ren and colleagues [145], which included 53 retrospective studies, for a total of 2,100,587 patients: hypertensive patients treated with ACE inhibitor/ARBs showed a lower risk of severe disease (odds ratio 0.81, 95% CI 0.66–0.99, *p* < 0.05) and death (odds ratio 0.81, 95% CI 0.66–0.99, *p* < 0.05), while no difference in COVID-19 incidence was observed.

Nonetheless, many of the above-mentioned studies presented different kinds of biases, and all of them were affected by their retrospective nature.

Unfortunately, at this date, there is only one randomized controlled trial (RCT) on the subject, published on The Lancet in January 2021 [146]. In this study, that included 175 hospitalized patients who were prescribed ACEI or ARB therapy as an outpatient before the hospital admission, participants were randomly allocated to continuation or discontinuation of such therapy. No difference between the two groups was found in a composite endpoint including days to death during the hospitalization, days on invasive mechanical ventilation or extracorporeal membrane oxygenation, days on renal replacement therapy or inotropic or vasopressor therapy and area under the curve (AUC) of a modified SOFA score.

## 7. Future Directions

Given the important role it likely plays in the pathogenesis of COVID-19, the RAAS system represents a promising therapeutic target.

As we have seen, there is sufficient evidence to affirm that ACE inhibitors and sartans, classic RAAS targeting drugs, can be used safely in patients with COVID-19; however, more data is needed to understand if they can be beneficial. Currently, several prospective randomized trials on the use of ACEI or ARB as a therapy for COVID-19 patients are underway (e.g., NCT04335786, NCT04311177, NCT04328012).

However, if it is true that upregulation of ACE2 activity by the virus contributes to COVID-19 pathogenesis, targeting only the canonical ACE-AT2-AT2R1 axis might not be enough and that could explain the mild results obtained so far. Thus, on these bases, it could be reasonable trying to pharmacologically inhibit both arms of RAAS.

Several molecules have been developed to specifically inhibit human ACE2, and some of them have been widely used in mouse/rat models [27]. Among them, MLN-4760 seems of particular interest, as it was not associated to any significant adverse effect in animal models [147,148,149,150,151,152], can be administer by several routes (including inhalation) and has shown to retain its inhibitory effects on soluble ACE2 bound to spike proteins [52], indicating that it is able to bind and inhibit ACE2 activity regardless ACE2 binding to SARS-CoV-2 particles (Figure 1).

Another possibility, is to act upstream on the RAAS pathways using a direct renin inhibitor, such as the FDA approved aliskiren, to reduce production of AT1, thus subtracting the fuel for both ACE and ACE2 pathways (Figure 1) [27,153].

Last but not least, an intriguing alternative approach is represented by zinc-chelating agents (Figure 1): as ACE, ACE2 and even ADAM17 are all zinc-metalloprotease, administration of zinc chelants could at the same time mitigate the hyperactivation of both arms of the RAAS and counteract the shedding of ACE2 induced by the virus [27,45,46,69]. On this regard, bismuth compounds (which have been widely used in humans for Helicobacter pylori treatment) have shown to exhibit potent anti-SARS-CoV-2 activity in vitro and in vivo [154,155]. In fact, bismuth compounds, in vitro, inhibit SARS-CoV-2 helicase via an irreversible displacement of zinc ions, but it’s not excluded that bismuth-mediated zinc displacement of other metalloproteases may also contribute to produce beneficial effect in vivo. Similarly, the cation chelator CaNa2EDTA has been used in humans as a treatment for heavy metal poisoning [89,156] and has been shown to be able to inhibit ACE2 activity [156].
